# The yin/yang balance of the MHC-self*-*immunopeptidome

**DOI:** 10.3389/fimmu.2022.1035363

**Published:** 2022-11-02

**Authors:** Laura Santambrogio, Alessandra Franco

**Affiliations:** ^1^ Department of Radiation Oncology, Physiology and Biophysics, Englander Institute of Precision Medicine, Weill Cornell Medicine, New York, NY, United States; ^2^ University of California San Diego School of Medicine, Department of Pediatrics, La Jolla, CA, United States

**Keywords:** MHC class I, MHC class II, immune tolerance, regulatory T cells, antigen processing, antigen presentation, peptides

## Abstract

The MHC-*self* immunopeptidome of professional antigen presenting cells is a cognate ligand for the TCRs expressed on both conventional and thymic-derived natural regulatory T cells. In regulatory T cells, the TCR signaling associated with MHC-peptide recognition induces antigen specific as well as bystander immunosuppression. On the other hand, TCR activation of conventional T cells is associated with protective immunity. As such the peripheral T cell repertoire is populated by a number of T cells with different phenotypes and different TCRs, which can recognize the same MHC-self-peptide complex, resulting in opposite immunological outcomes. This article summarizes what is known about regulatory and conventional T cell recognition of the MHC-self-immunopeptidome at steady state and in inflammatory conditions associated with increased T and B cell self-reactivity, discussing how changes in the MHC-ligandome including epitope copy number and post-translational modifications can tilt the balance toward the expansion of pro-inflammatory or regulatory T cells.

## Introduction

The main role of the thymus is to generate functionally competent T cells, which respond to pathogens but are tolerant to self-antigens (1). During T cell development the T cell receptor (TCR) of maturing T cells interacts with the MHC-self-peptides presented by different thymic antigen presenting cells. The TCR-MHC-peptide interaction occurs within a great range of affinities and the functional outcome of these interactions results in positive and negative T cell selection of both conventional (T_conv_) or regulatory (T_reg_) T cells (2–4). Overall a “weak” TCR signal is conducive to positive selection, and the majority of T_conv_ cells are generated within this domain of affinities (3, 4). On the other hand, murine models have established that T_reg_ are generated from a niche of T cells rescued from the negatively selected pool (5, 6).

Once they populate the periphery T_conv_ cells shape the immune responses from immunity to pathogens, to the cytotoxic engagement of tumor cells (7, 8). Regulatory T cells (T_reg_) are pivotal to immune homeostasis, implementing immune tolerance to self and symbiotic commensal, monitoring immune responses, and maintaining tissue homeostasis (8–10). Natural T_reg_ (nT_reg_) are generated in the thymus and peripheral T_reg_ (pT_reg_) are generated in the periphery, following differentiation from conventional naïve CD4^+^ T cells when exposed to suboptimal antigen concentration and repeated stimulations, or the commensal microbiome (11). nT_reg_ are strategically located in the T and B cell areas of secondary lymphatic organs where they can control both adaptive arms of the immune response (8). To suppress/regulate immune responses T_reg_ rely on cell-surface inhibitors such as CTLA4 and PD-1 as well as the secretion of inhibitory cytokines including IL-10 and TGF-β (8, 12). Additionally, by sequestering IL-2, they control T_conv_ proliferation and clonal expansion (13).

As for T_conv_, the TCR engagement by the cognate MHC-peptide ligand is also pivotal for T_regs_ differentiation in the thymus, where the affinity of their TCR for MHC-self-peptides rescues them from clonal deletion and set them apart from T_conv_ (14). Similarly, in the periphery, the tonic signal of T_reg_-TCR engagement is necessary to maintain their expansion and insure their suppressor function (14).

In the last decade, diverse T_reg_ sub-phenotypes as well as their TCR repertoires have been investigated, giving insight into T_reg_ heterogenicity as well as contributing to the notion that the T_reg_ TCR repertoire is as broad as that of T_conv_ (9, 15–17).

However, an area that is very much under-investigated is the fine antigen specificity and the MHC-restricted immunopeptidome recognized by T_reg_ and how much this overlap, or is set apart from the MHC-restricted repertoire recognized by T_conv_. More importantly, further research is required to examine how the balance between T_conv_ and T_reg_ recognition of the same MHC-self-peptide complex shape immune responses.

This review summarizes what is known about T_reg_ self-antigen recognition and the related MHC-immunopeptidome and its interplay with the MHC-immunopeptidome recognized by T_conv_ at steady state and in inflammatory acute and chronic conditions associated with increased T and B cell autoreactivity.

## Recognition of the MHC-self-peptidome: Conventional and regulatory T cells

T cell recognition of cognate MHC-peptide ligands is not an on-off binary switch, since the TCR can “sense” differences between optimal and suboptimal ligands and signal accordingly (18). Agonist peptides, even in low nanomolar concentrations, can stimulate proliferation and effector functions (cytokine production, cytotoxic responses) in CD4^+^ and CD8^+^ T cells. Partial agonists require a higher concentration to induce the same T cell responses and secretion of effector lymphokines, antagonist peptides specifically inhibit the response(s) that can be induced by an agonist *via* single amino acid substitutions of major TCR contacts (18). As such, the α/β T-cell receptor present on CD4^+^ and CD8^+^ T cells can distinguish subtle structural variations in the MHC-peptide conformation and translate the affinity/avidity of cognate ligand recognition into distinct T cell responses. The ability of the TCR to conduce distinct signals following peptide-MHC engagement plays a pivotal role in directing T_conv_ and T_reg_ development. Indeed, in the last decade it has become apparent that, in the thymus, the same MHC-peptide complex can induce thymocyte deletion and generate T_conv_ and nT_reg_ (19).

During thymic selection TCRs with high MHC-peptide affinity can generate T_reg_, which is selected for highly stringent recognition of an agonist MHC-*self*-peptide. At the same time, T cells with low/medium affinity for self-peptides undergo positive selection generating T_conv_. Similarly, in the periphery, the same MHC-peptide complex can provide the tonic signal necessary to maintain a peripheral T cell repertoire composed of conventional/effectors and thymic-derived nT_reg_ (20). On the other hand, repeated antigen stimulations can induce the switching of naïve T cells and sub-optimally stimulated pro-inflammatory T cells into pT_reg_ (21).

The peripheral T cell repertoire is populated by a number of T cells with different functional phenotypes (T_conv_ and T_reg_) and different TCRs that can recognize the same MHC-self-peptide complex with different affinities and generate pro-inflammatory or regulatory immune responses (22, 23).

T_reg_ directly controls around 30% of the autoreactive T cell population from converting into pathogenic effectors (22, 24). At steady state, pT_reg_ by having a TCR with higher affinity for the same MHC-peptide complex, as compared to T_conv_, are likely to require lower antigen concentration and by default, a lower MHC-epitope copy number to be activated. As such, pT_reg_ can directly suppress the immune response of T_conv_ specific for the same MHC-peptide (25). However, T_reg_ and nT_reg_ in particular, can also effectively suppress T_conv_ specific for a different MHC-peptide complex through secretion of anti-inflammatory cytokines. However, for this non-cognate suppression to occur, nTreg need to be activated by the recognition of their cognate MHC-peptide ligand for TCR signaling and optimal activation and function (26).

In pathological conditions, changes in the dendritic cell MHC-antigen processing and presentation machinery can affect the selection, affinity, composition, and epitope copy number of the MHC-ligandome (27–30). This is associated with up-regulation in the costimulatory molecule and increased adjuvanticity of the tissue microenvironment, which can tilt the T_reg_/T_conv_ balance and favor autoreactivity (30, 31).

We and others have observed and demonstrated that in multiple chronic inflammatory and dysmetabolic conditions there is increased T and B cell autoreactivity (29, 30, 32–37). For example, in cardiovascular disorders elevated circulating levels of autoantibodies targeting cardiac or vascular (29) proteins such as troponin I3, cardiac type (TNNI3) (38), oxidized apolipoproteins (39), as well as ubiquitous inflammation-associated proteins such as heat shock proteins (HSPs) (40) have been reported. Similarly, autoreactive T cells and antibodies specific to several cytosolic self-antigens including glutamate decarboxylase 1 (GAD1), islet cell autoantigen 1 (ICA1), INSM transcriptional repressor 1 (INSM1) and solute carrier family 30 member 8 (SLC30A8) have been reported in the serum of patients with metabolic syndrome and type 2 Diabetes (T2D) (41).

Recently, we demonstrated that in Type 2 Diabetes (T2D), the chronic inflammatory environment increased the MHC II presentation of peptides derived from stress-associated proteins by local dendritic cells, including protein disulfate isomerase-3 (PDIA3) (30). Stress-related responses also induced PDIA3 translocation at the plasma membrane, facilitating auto-Ab recognition (30). Ultimately, the increased presence of the MHC II-restricted PDIA3 peptide and increased titers of IgG2b and IgG3 anti-PDIA3 antibodies with cytotoxic activity aggravated liver tissue damage by tilting the balance from tolerance to autoreactivity (30). The pathogenic connotation of anti-PDIA3 immune responses was evident following the passive transfer of cognate CD4^+^ T cells and antibodies that induced hepatocyte cytotoxicity (30). Similarly, it was demonstrated that an I-Ab-restricted Apolipoprotein B peptide (ApoB) could induce *in vivo* T_reg_ or T_conv_ inflammatory responses under opposite environmental conditions (29, 42, 43).

Since both PDIA3 and ApoB peptides are recognized by both T_conv_ and T_reg_ the stoichiometry of their MHC presentation contributed to tilt the balance towards tolerance or inflammation. Under physiological conditions, around 0.4 femtomoles of PDIA3 peptide and 0.05 femtomoles of ApoB peptide were presented by I-Ab, however in dysmetabolic conditions, due to a high fat, high sucrose diet, a 40% increase in I-Ab presentation of both PDIA3 and ApoB epitopes was observed (29, 30). We reasoned that at steady state T_reg_ requires lower amounts of *self*-antigens, or MHC-epitope copy number to be activated, due to their higher affinity TCR, as compared to the T_conv_ TCR specific for the same MHC-peptide complex (9, 14, 25). However, during acute and chronic inflammatory conditions associated with immunogenic cell death, increased antigen availability and MHC-epitope copy number, associated with an environment rich in pro-inflammatory cytokines and damage-associated-molecular pattern (DAMPs) can activate T_conv_ even if the same MHC-peptide complex is recognized by T_reg_ and overcome their suppression (29–31).

## Translational therapeutic applications: Conventional and regulatory T cell balance

Different strategies in early clinical trials have been designed to optimize T_reg_ expansion to suppress autoreactive T cells and autoimmunity even when the disease is in progress (44, 45). Insulin-derived T_reg_-activating peptides have been mapped both in NOD mice and T1D humans (46, 47); both peptides have been tested in pre-clinical studies. Initial results indicate that the islet cell function was preserved in patients receiving the peptide treatment, as compared to the no-treatment group (47). In another study, Hsp70-derived peptides have been shown to induce T_reg_ cells in clinical studies of Type 1 Diabetes (T1D) (48) and rheumatoid arthritis (RA) (49). Finally, early phase clinical trials with autoantigen specific therapy in Multiple Sclerosis have also shown promising results in inducing T_reg_-mediated immune suppression (50–52).

When analyzed for anaphylactic reactions it appears that both dosage, timing, and biophysical properties of MHC-peptide binding play a role. For example, the development of the B9-23 insulin peptide for therapeutic purposes indicated that prolonged administration of the peptide induced anaphylaxis in NOD mice (53). Subsequent MHC-peptide binding studies indicated that MHC-peptide affinity/stability favored T_conv_ activation over T_reg_ (54). On the other hand, self-peptides administered to over 1000 lupus-prone mice did not indicate any adverse reactions. As shown, histone peptides generated T_reg_ that induced TGF-β-mediated suppression without the Th2 skewing associated with the allergic reactions seen in other autoimmune diseases, such as experimental autoimmune encephalomyelitis (EAE), Multiple Sclerosis (MS) and T1D in NOD mice (47, 55–57).

The balance between T_conv_/T_reg_ MHC-self peptide recognition can also be tilted by protein/peptide post-translational modifications. It has long been recognized that in conditions associated with chronic inflammation or in dysmetabolic conditions an increase in oxidative stress, hyperglycemia and hyperlipidemia contribute to non-enzymatic protein oxidation, glycation, and lipoxidation (58–66). The protein post-translational modifications (PTMs) are carried over during endosomal/proteasomal processing and MHC-loading, generating an immunopeptidome where some of the amino acids are modified by the bulky oxidative residues (29, 63). Since these peptides are not presented in the thymus, they may not engage T_reg_. At the same T_conv_ could be activated by the PTM-modified peptides (67, 68). This mechanism has been extensively reported for citrullinated peptides in RA (69, 70), oxidized ApoB peptide in atherosclerosis, and cardiovascular diseases (71), nitrosylated peptides in degenerative brain disorders (72), acetylated and citrullinated peptides in lupus (73) and deamidated peptides in melanoma-associated immunogenicity (74).

Albeit the majority of MHC-self peptides are cognate ligands for both T_conv_ and T_reg_, during the last decade few studies have pinpointed MHC-restricted epitopes within the human/mouse self-proteome which strongly induce nT_reg_. Among those, the best characterized have been epitopes processed from histones, albumin(s), and immunoglobulins; likely, due to their high abundance, differently from most tissue specific self-antigens, they are presented in the thymus, at high copy number, during nT_reg_ development.

Histone epitopes are mostly generated from the processing of nucleosomes of apoptotic cells; as known apoptotic cells are physiologically cleared by the immune system without activating an immune response (75). Indeed, cellular apoptosis is a daily occurrence in different organs, particularly in primary lymphoid organs such as the thymus and bone marrow. Peptides derived from apoptotic cells are presented in MHC I and MHC II restriction to educate maturing T and B cells and, histone peptides have been shown to generate CD4^+^ and CD8^+^ T_reg_ (56, 76–80).

The yin/yang balance between immunogenic and tolerogenic responses to the self*-*peptidome can be best visualized in lupus where several immunogenic self-peptides, derived from nucleosomal histones, inducing effector T cells in lupus nephritis have been mapped (78, 81, 82). The same peptides, when administered in low doses (1 µg, sub-cutaneous every 2 weeks) induced a low-dose tolerance which effectively lowered autoantibody levels, blocked nephritis progression, and markedly diminished inflammatory cell infiltration in the kidneys (77). The low dose antigen therapy was shown to induce regulatory T cell subsets with a CD8^+^ CD25^high^, and CD4^+^CD25^high^ phenotype, which both lowered IFN-γ production by autoreactive pro-inflammatory T cells and induced TGF-β secretion in response to the histone epitopes. Importantly, the T_reg_-induced suppression was maintained *in vivo* following passive cell transfer where even low dose tolerance with one self peptide epitope could halt the lupus progression (56). Splenic dendritic cells (DC), but not B cells or macrophages were the antigen presenting cells (APC) responsible for the antigen presentation to T_reg_ and for the expansion of epitope-specific and cross-reactive T_reg_, that suppressed lupus effector T helper (Th)1 and Th17 cells (56). The peptide-induced T_reg_ in PBMC from patients with lupus depended on TGFβ/ALK-5/pSmad 2/3 signaling. Interestingly the DC pulsed with the tolerogenic histone peptide showed a decreased inflammatory phenotype with down-regulation of CD80, CD86, and CD40 co-stimulatory molecules and decreased MHC class II surface expression, as compared to non-peptide pulsed DC (55, 56).

In a murine model of lupus, immune tolerance could be induced by nasal administration of very low amounts of pathogenic self-peptides leading to the expansion of T cells that secrete TGF-β and low amounts of pro-inflammatory cytokines (55, 83). Histone-based therapy also induced CD8^+^ T_reg_ cells to stably express FOXP3 and increased levels of CTLA-4, CD103, PD-1, PD-L1, and LAP, when compared to CD8^+^ T cells from the same patients before undergoing kidney transplant (84). These cells were considerably more potent in their suppressive activity as compared to the CD4^+^CD25^high^ T_reg_ that appeared during clinical “remission” in lupus patients (84–86). Similar responses were observed upon administration of other known lupus autoantigens such as small nuclear ribonucleoproteins and nuclear ribonucleoproteins (87). To summarize, the T_reg_ cells induced by the histone epitopes directly and indirectly suppressed innate immune cells (DC), T cells, and B cells involved in the pathogenic autoimmune response.

Additionally, histone peptides have also been shown to have immunosuppressive activity by activating a subtype of T_reg,_ named follicular regulatory T cells (T_frs_) (CXCR5^high^ PD-1^high^ and FoxP3^+^), which are located in B cell follicles of secondary lymphoid organs. T_frs_ play pivotal roles in regulating B cell responses and inhibiting the development of auto-Ab (88–91). Histones and nuclear proteins have been shown to induce T_frs_ expansion and up-regulation of immunosuppressive genes (92). Once activated T_frs_ promote inhibition of germinal center B cells, in particular B cells with a BCR specificity towards nuclear proteins, indicative of antigen-specific T_reg_ suppression (93). However, the histone epitopes can induce T_reg_ that suppresses both antigen specific as well as bystander T and B cells, altogether regulating pathogenic immune responses (93).

The second set of well-characterized T_reg_ epitopes that induced both thymic and peripheral T_reg_ (94–98) were IgG peptides, deriving from the processing of the Fc heavy chain constant region (99–101). *Ex vivo* elegant studies have shown that in children with Kawasaki disease (KD), an acute pediatric vasculitis of the coronary arteries, IgG administered intravenously (IVIG) were mostly internalized by receptor-mediated phagocytosis, Fcγ receptor (R) II and to a lesser extent FcγRIII, by two myeloid tolerogenic DC populations, CD14^+^ CDC2 and ILT-4^+^ CD4^+^ tmDC. Fc processed peptides induced T_reg_ expansion and IL-10 production by both T_reg_ and the presenting DC, indicating the role of both innate and adaptive tolerogenic responses following Fc heavy chain constant region presentation (97–99, 101, 102).

In a different set of studies, IgG peptides, when administered prior to diabetes insurgence in NOD mice, completely abrogated the development of the disease and, when administered after diabetes insurgence suppressed the disease progression (103), even when injected together with insulin immunogenic peptides (103, 104). Finally, IgG+ B cells have been shown to present immunodominant Fc peptides to nT_reg_ *via* a unique antigen processing of the surface IgG that differs from the exogenous up-take of IgG by tolerogenic DC. Of interest, the most tolerogenic Fc peptides recognized by T_reg_ bind multiple MHC class II alleles, including DR, DP, and DQ, and share the same sequences in healthy donors and RA subjects (101).

## Conclusions

Analyses of the MHC-ligandome recognized by T_reg_ and T_conv_ are an important endeavor, due to the pivotal role of both cells in immune responses. Even though there is not yet extensive literature on the subject, it appears, as expected, that no MHC-peptide is uniquely recognized by either T_reg_ or T_conv_. Indeed, for the peptides analyzed in depth so far, it appears that in the thymus the presented MHC-immunopeptidome generates a T cell repertoire that comprises both T_reg_ and T_conv_. At steady states, it is likely that T_reg_ exercise control over T_conv_ responses due to their thymically-selected high affinity TCR, which requires a lower peptide concentration for T_reg_ activation (1). This low-dose tolerance is likely how tolerance to tissue-specific antigens, notoriously present in low amounts, is generated (50, 52, 56). The same mechanism is exploited by “low-dose” peptide therapies used to activate T_reg_ in many autoimmune diseases. However, T_reg_ also recognizes several MHC-epitopes normally presented at high MHC-copy numbers, such as nuclear proteins, immunoglobulins, and albumins. All these antigens have also been shown to effectively activate T_reg_ and strongly down-modulate inflammation and autoimmunity, as seen in several clinical trials. It has been postulated that this “high-dose tolerance” may be important in maintaining a pool of T_reg,_ easily activated by abundant antigens, which down-regulate immune responses through by-standard suppression and secretion of anti-inflammatory cytokines (97, 102).

Further work is necessary to determine the T_reg_/T_conv_ antigen specificity and degeneracy, the role of low dose *vs* high dose tolerance in relationship to T_reg_/T_conv_ generation, TCR affinity avidity, and signaling, as well as the contribution of MHC-epitope copy number to the activation of either T cells and finally, the role of tissue microenvironment in keeping or tilting the T_reg_/T_conv_ balance.

## Author contributions

All authors contributed to the article and approved the submitted version.

## Conflict of interest

The authors declare that the research was conducted in the absence of any commercial or financial relationships that could be construed as a potential conflict of interest.

## Publisher’s note

All claims expressed in this article are solely those of the authors and do not necessarily represent those of their affiliated organizations, or those of the publisher, the editors and the reviewers. Any product that may be evaluated in this article, or claim that may be made by its manufacturer, is not guaranteed or endorsed by the publisher.
